# Inhibition of ferrochelatase impairs vascular eNOS/NO and sGC/cGMP signaling

**DOI:** 10.1371/journal.pone.0200307

**Published:** 2018-07-09

**Authors:** Bin Zhang, Norah Alruwaili, Sharath Kandhi, Wensheng Deng, An Huang, Michael S. Wolin, Dong Sun

**Affiliations:** Departments of Physiology, New York Medical College, Valhalla, New York, United States of America; Universitat Regensburg, GERMANY

## Abstract

Ferrochelatase (FECH) is an enzyme necessary for heme synthesis, which is essential for maintaining normal functions of endothelial nitric oxide synthase (eNOS) and soluble guanylyl cyclase (sGC). We tested the hypothesis that inhibition of vascular FECH to attenuate heme synthesis downregulates eNOS and sGC expression, resulting in impaired NO/cGMP-dependent relaxation. To this end, isolated bovine coronary arteries (BCAs) were *in vitro* incubated without (as controls) or with N-methyl protoporphyrin (NMPP; 10^−5^–10^-7^M; a selective FECH antagonist) for 24 and 72 hours respectively. Tissue FECH activity, heme, nitrite/NO and superoxide levels were sequentially measured. Protein expression of FECH, eNOS and sGC was detected by western blot analysis. Vascular responses to various vasoactive agents were evaluated via isometric tension studies. Treatment of BCAs with NMPP initiated a time- and dose-dependent attenuation of FECH activity without changes in its protein expression, followed by significant reduction in the heme level. Moreover, ACh-induced relaxation and ACh-stimulated release of NO were significant reduced, associated with suppression of eNOS protein expression in NMPP-treated groups. Decreased relaxation to NO donor spermine-NONOate reached the statistical significance in BCAs incubated with NMPP for 72 hours, concomitantly with downregulation of sGCβ1 expression that was independent of heat shock protein 90 (HSP90), nor did it significantly affect BCA relaxation caused by BAY 58–2667 that activates sGC in the heme-deficiency. Neither vascular responses to non-NO/sGC-mediators nor production of superoxide was affected by NMPP-treatment. In conclusion, deletion of vascular heme production via inhibiting FECH elicits downregulation of eNOS and sGC expression, leading to an impaired NO-mediated relaxation in an oxidative stress-independent manner.

## Introduction

Heme is an essential prosthetic group for hemoproteins that are involved in multiple physiological processes, including oxygen transport and storage, oxidases and antioxidant defenses, mitochondrial respiration and electron transport, drug metabolism and protein biosynthesis, etc. [1,2]. Ferrochelatase (FECH) is an important rate-limiting enzyme in heme biosynthesis, during which, FECH operates the last enzymatic reaction by inserting ferrous iron (Fe^2+^) into protophorphyrin IX (PpIX) [3–5]. It has been demonstrated that increases in oxidative stress, as a general consequence of a variety of pathological procedures, impair FECH/heme regulatory processes via first, disrupting iron-sulfur clusters on FECH [6,7] to decrease its activity [8], followed by reduction of heme synthesis. Second, the oxidation of heme present in some heme-containing enzymes inactivates their enzymatic activity. In this context, oxidation of soluble guanylyl cyclase (sGC) via converting heme from the ferrous (Fe^2+^) to ferric (Fe^3+^) form has been documented as a key mechanism responsible for the peroxynitrite-dependent attenuation of sGC activity [9–11]. Furthermore, oxidation of the sGC-heme causes a loss of NO-stimulated cGMP production, associated with a proteolytic depletion of sGC in some vascular diseases [12]. Heme-containing enzymes of endothelial nitric oxide synthase (eNOS) and sGC are crucially implicated in the regulation of cGMP-controlled processes by NO, a pathway that physiologically controls many cellular functions in the cardiovascular system [2,13]. Since there are differences in sensitivities to the impact of heme deficiencies on the expression of some hemoproteins [14,15], changes in heme biosyntheses are hypothesized to significantly interrupt components of eNOS/sGC/cGMP signal transduction. Moreover, we have demonstrated that *in vivo* treatment of mice with δ-aminolevulinic acid (ALA), a heme-synthesis precursor that promotes PpIX-elicited activation of sGC, attenuated hypoxia-induced pulmonary hypertension via preserving sGC/cGMP-dependent vasodilation [16,17]. Additionally, impaired NO-mediated vasodilator responses of bovine coronary arteries (BCAs), as a function of angiotensin II (Ang II)-induced up regulation of mitochondrial superoxide elicited disruption of FECH activity, a response that was normalized by treatment of BCAs with ALA [18]. Moreover, miR-210 was reported to be able to compromise cardiac heme production by targeting and inhibiting FECH [5], the study however, did not evaluate responsible functional changes. Thus, while disrupting heme synthesis by oxidative stress to alter sGC/cGMP signaling has been has been documented by our previous study [18], evidence is lacking indicating the heme depletion-caused an interruption of eNOS synthesis/expression, as a function of directly inhibiting FECH to deplete heme. While an *in vivo* inhibition of heme synthesis initiated significant reductions in systemic nitrite/nitrate excretion and renal NOS activity of rats [19], it is worth noting that the *in vivo* intervention of heme synthesis can initiate systemic changes in multiple signal pathways and/or molecules involved, such as oxidations and anti-oxidative defense or iron-sulfur cluster scaffold proteins and components of the electron transport chain, etc., all of which may not necessarily be direct consequences of FECH inhibition or heme loss, but rather a biological complexity that links multiple events operating in concert to elicit responses observed. Additionally, most previous studies were focused on oxidative stress that disrupts heme/NO/sGC/cGMP signaling involving both FECH/heme-dependent and -independent mechanisms. To this end, the present study aimed to test the hypothesis that the inhibition of FECH, followed by heme deficiency [14] directly downregulates eNOS and sGC expression to alter NO/cGMP-mediated responses.

## Materials and methods

### Vessel culture

Freshly isolated bovine hearts were kept in ice-cold PBS and transported to the laboratory within three hours from a local slaughterhouse (Cohen Max Insel Animal Organs & Tissues for Research Inc, Livingston, NJ). BCAs were isolated from the branches of left anterior descending arteries and cut into rings of 2 mm length under a dissecting microscope. BCAs were then placed in a 12-well dish filled with DMEM (Cellgro), supplemented with 10% fetal bovine serum (v/v) and 1% antibiotics (Antimycin solution 100x), and incubated in the absence (as controls) and presence of different concentrations of N-methyl protoporphyrin (NMPP; 10^−7^–10^-5^M; a selective FECH antagonist) (Frontier Scientific, Logan, UT, USA) under a 5% CO_2_ atmosphere at 37°C for 24 and 72 hours respectively. DMEM containing NMPP was prepared fresh and replaced every 12 hours.

### Measurement of ferrochelatase (FECH) activity by HPLC-based assay

BCA rings were pulverized in liquid nitrogen. The crushed samples were mixed with a sample buffer (0.25 M Tris·HCl buffer, pH 8.2, containing 1% Triton X-100 and 1.75 mM of palmitic acid) in a concentration of 1 mg tissue per 100 μl buffer. Samples were incubated on ice for 30 min and then centrifuged at 1000 g for 5 min. The supernatant was collected and protein concentration was measured by Bio-Rad protein assay kit (Bio-Rad Laboratories, Hercules, CA). 20 μl samples with protein concentration of 0.5 μg/μl were mixed with 5 μl of protoporphyrin IX (PpIX; 250 μM). The reaction was initiated by adding 5 μl 200 μM zinc acetate and incubated at 37°C for 1 hour. After that, 170 μl dimethyl sulfoxide-methanol (30:70) solution was added to stop the reaction. HPLC measurement of zinc protoporghyrin (ZnPpIX) was used as an indicative of FECH activity with a Beckman ultrasphere C18 column (5 μm, 150 × 2 mm), a Jasco FP-1520 fluorescence detector, and 0.5 ml/min acetone-methanol-water-formic acid (560:240:200:2) as the mobile phase. ZnPpIX was detected based on the amount of fluorescence observed with excitation and emission wavelengths of 415 and 580 nm, respectively. Standard curves of ZnPpIX (0.1–5 picomole) were generated using the sample buffer as a vehicle and used to calculate ZnPpIX formation, expressed as picomoles per mg protein per minute.

### Measurement of vascular heme

As described previously [20], BCA rings were washed several times with Krebs solution and homogenized in 20 mM MOPS and 250mM sucrose buffer. Homogenates were centrifuged at 2,000 g for 5 min, and supernatant were assayed for protein content. 50 μl obtained from a 5 mg protein/ml supernatant were quantified for heme content using the QuantiChrom heme assay kit (BioAssay Systems, Hayward, CA, USA). The absorbance at 400 nm was detected by Synergy HT spectrophotometer (BIOTEK, Winooski, Vermont, USA). BCA heme levels were reported as nanomoles per mg protein.

### Western blot analysis

Frozen BCAs were pulverized in liquid N_2_. Equal amounts of total protein (25 μg) extracted from samples were loaded on and separated by a 10% SDS-PAGE gel and transferred to a PVDF membrane. The membrane was probed with specific primary antibodies for FECH (Abcam, MA), eNOS (Santa Cruz Biotechnology, CA), sGCβ1 (Sigma, MO), vasodilator-stimulated protein (VASP), phospho-VASP serine 239 (p-VASP) and heat shock protein 90 (HSP90) (Cell Signaling Technology, Danvers, MA), followed by appropriate secondary antibodies conjugated with horseradish peroxidase. Specific bands were visualized with a chemiluminescence kit and normalized to GAPDH. For mitochondrial FECH expression, the mitochondrial protein of voltage-dependent anion channel (VDAC) was used as loading control. The X-ray film was scanned into a computer and band densitometry was digitalized with UN-SCAN-IT software.

### Isometric tension experiments

The method was described in detail previously [21]. Briefly, isolated BCA rings were mounted on Danish myograph setups (DMT620M; Danish Myo Technology, Aarhus, Denmark) using 200 μm stainless steel pins and perfused with physiological salt solution (PSS) buffered with 95% air and 5% CO_2_ at 37°C. The internal diameter and circumference of each ring was determined by the length of the ring and the known geometry of pins when the ring was stretched to a level that generated the least stretching force. The average width (1.69 ± 0.07 mm and 1.61± 0.06 mm for 24-h cultured groups, 1.55 ± 0.05 mm and 1.57± 0.06 mm for 72-h cultured groups) and diameter (612.2 ± 46.2 μm and 654.5 ± 46.6 μm for 24-h cultured groups, 575.3 ± 38.9 μm and 583.6 ± 41.4 μm for 72-h cultured groups) of rings were comparable in control and NMPP-treated rings. Based on the circumference, rings were further stretched in a stepwise manner to establish a length-tension relationship. Using the length-tension curve, a baseline force that was equivalent to a wall tension generated under 80 mmHg of intravascular pressure was calculated and applied to the rings. The average baseline force (23.9 ± 2.9 mN and 23.7 ± 2.8 mN for 24-h cultured rings, 20.6 ± 2.3 mN and 19.1 ± 2.7 mN for 72-h cultured rings) and corresponding diameter of stretched rings (1429.4 ± 90.3 μm and 1406.0 ± 83.3 μm for 24-h cultured vessels, 1248.2 ± 109.5 μm and 1178.1 ± 119.3 μm for 72-h cultured vessels) were obtained from control and NMPP groups, respectively. All rings were equilibrated under baseline force in PSS for one hour. In all studies, arterial rings were depolarized with 122 mM KCl Krebs-bicarbonate buffer (high K^+^) to stabilize the reactivity of rings, followed by re-equilibration with Krebs-bicarbonate buffer for additional 30 min. Then, the vessels were precontracted with Krebs-bicarbonate containing 30 mM KCl (30K), and subsequently relaxed to increasing cumulative concentrations of spermine-NONOate (10^−8^–10^-5^M), isoproterenol (10^−9^–10^-5^M), acetylcholine (ACh, 10^−8^–10^-5^M) and Bay58-2627 (10^−9^–10^-5^M; Biovision Inc, Atlanta, GA) respectively. Data were reported as percentage relaxation of the developed force generated by 30K.

### Basal and stimulated release of NO/nitrite in BCAs

The method was described in detail previously [22]. Briefly, the BCA rings were incubated in 96-well plates with 200 μl Krebs buffer (pH 7.4) with one segment of BCA rings in each well for at 37°C for 1 hour. The buffer was collected to assess the baseline level of nitrite. After that, 200 μl Krebs buffer containing 10^-6^M ACh were added into each well, followed by incubation of the rings at 37°C for 1 hour. After that, the buffer was once again collected to assess the ACh-stimulated nitrite production. Nitrite formation was assessed using 2,3-diaminonaphthalene (DAN) and a HPLC/fluorescence detector-based assay to determine 1-(H)-naphthotriazole, a fluorescent product upon the reaction of nitrite and DAN. 20 μl DAN dissolved in N,N-dimethylformamide (5 mg/ml) and further diluted with 6 N HCl to 0.05 mg/mL, was added to 200 μl of the buffer and incubated for 10 min at room temperature. Then 20 μl of 10 N NaOH was added. After a centrifugation, 20 μl of supernatant was separated by an HPLC system (PU-2080 Plus; Jasco) with a C-18 reverse-phase column (Beckman Ultrasphere ODS, 5 μm, 4.6 × 250 mm). The mobile phase was composed of 35% acetonitrile and 50 mmol/L sodium phosphate buffer (pH 8.5) and run at a flow rate of 0.5 ml/min. The fluorescent signal of 1-(H)-naphthotriazole was detected at 375 nm (excitation) and 415 nm (emission) with a fluorescence detector (FP2020 Plus; Jasco). The standard curve of sodium nitrite (0–640 μmol/L) was generated and used to calculate nitrite formation in the sample, expressed as picomoles per centimeter squared of the internal surface of vessels. The internal surface of BCA rings were determined by mounting the rings on wire myograph apparatus and stretching rings to a point that generates the least forces.

### Vascular superoxide production

As described previously [23], were incubated with dihydroethidium (DHE, 10^−5^ mol/l) for one hour, during which the superoxide in the vessels reacted with DHE to form 2-hydroxyethidium (EOH), which was detected by HPLC/fluorescence detector. After incubation with DHE, the vessels were washed and homogenized in acetonitrile/water (1:1), and then centrifuged for 10 minutes. After centrifuging, the supernatant fraction was collected for HPLC analysis and the precipitate was used for protein measurement using Bio-Rad Protein Assay (Bio-Rad, Hercules, CA). The final concentration of superoxide in each sample was normalized to the protein contents of their corresponding vessels, and expressed as picomoles of superoxide (EOH) per microgram of protein.

### Statistics

Data are represented as mean ± SEM, and n refers to the number of hearts. Statistical analyses were performed using GraphPad Prism 6 software (Graph Pad Software Inc., San Diego, CA, USA). Two-way ANOVA was used to compare dose-dependent vasodilator responses between the control and NMPP-treated groups. Student’s t-test was used to compare the difference between two groups. Statistical significance was accepted at a level of p< 0.05.

## Results

### Inhibition of FECH decreases heme content in BCAs

**Fig 1:**
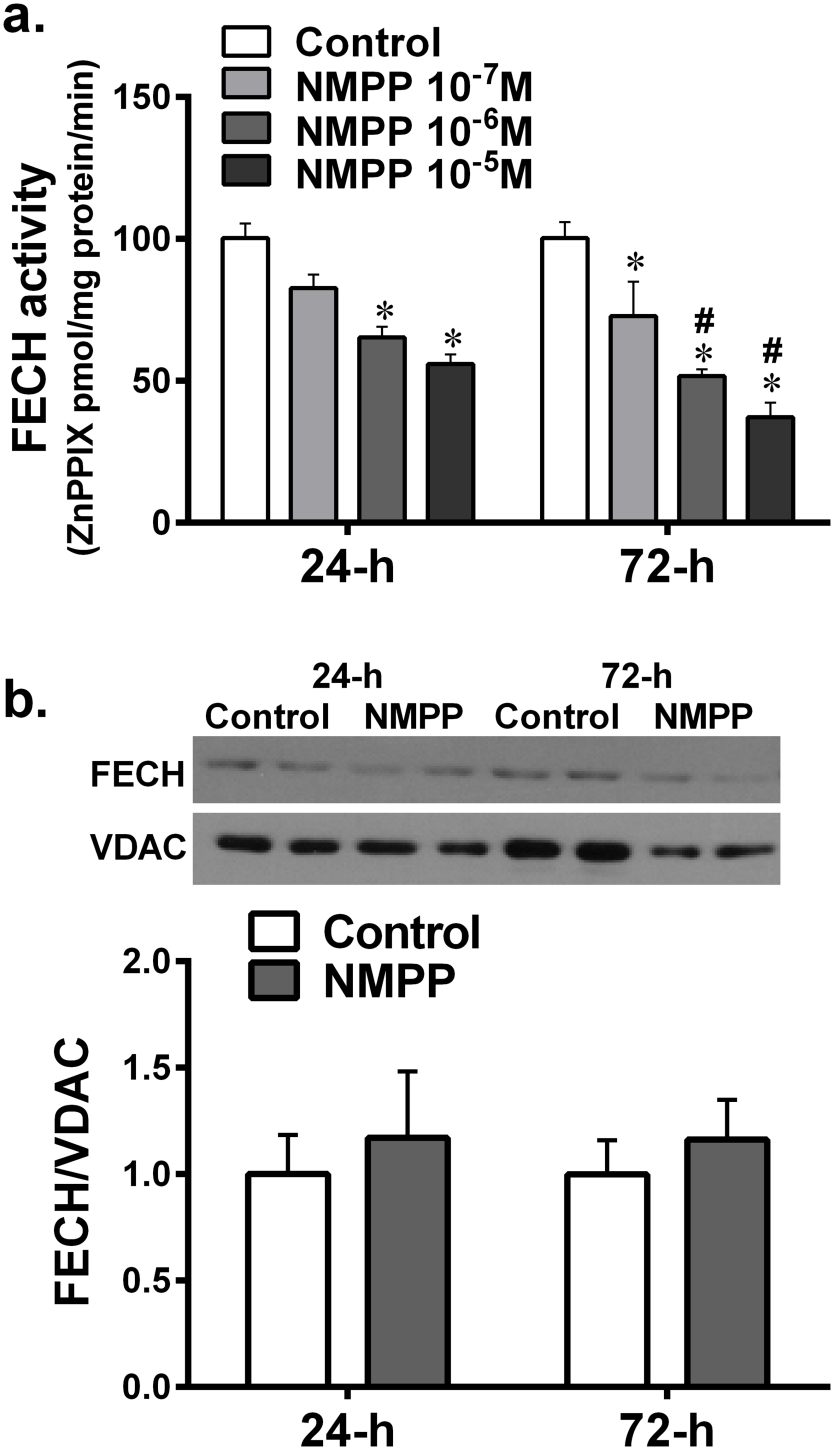
(a) Changes in vascular ferrochelatase (FECH) activity in isolated bovine coronary arteries (BCAs) incubated with difference doses of N-methyl protoporphyrin (NMPP;10^−7^, 10^−6^ and 10^-5^M) for 24 (24-h) and 72 hours (72-h), respectively (n = 6–10). *significant difference from their controls. #significant difference from 24-h group. (b) Original and summarized data for FECH protein expression, normalized to voltage-dependent anion channel (VDAC) in isolated BCAs incubated with 10^-6^M NMPP for 24 and 72 hours respectively (n = 3 blots).

**Fig 2:**
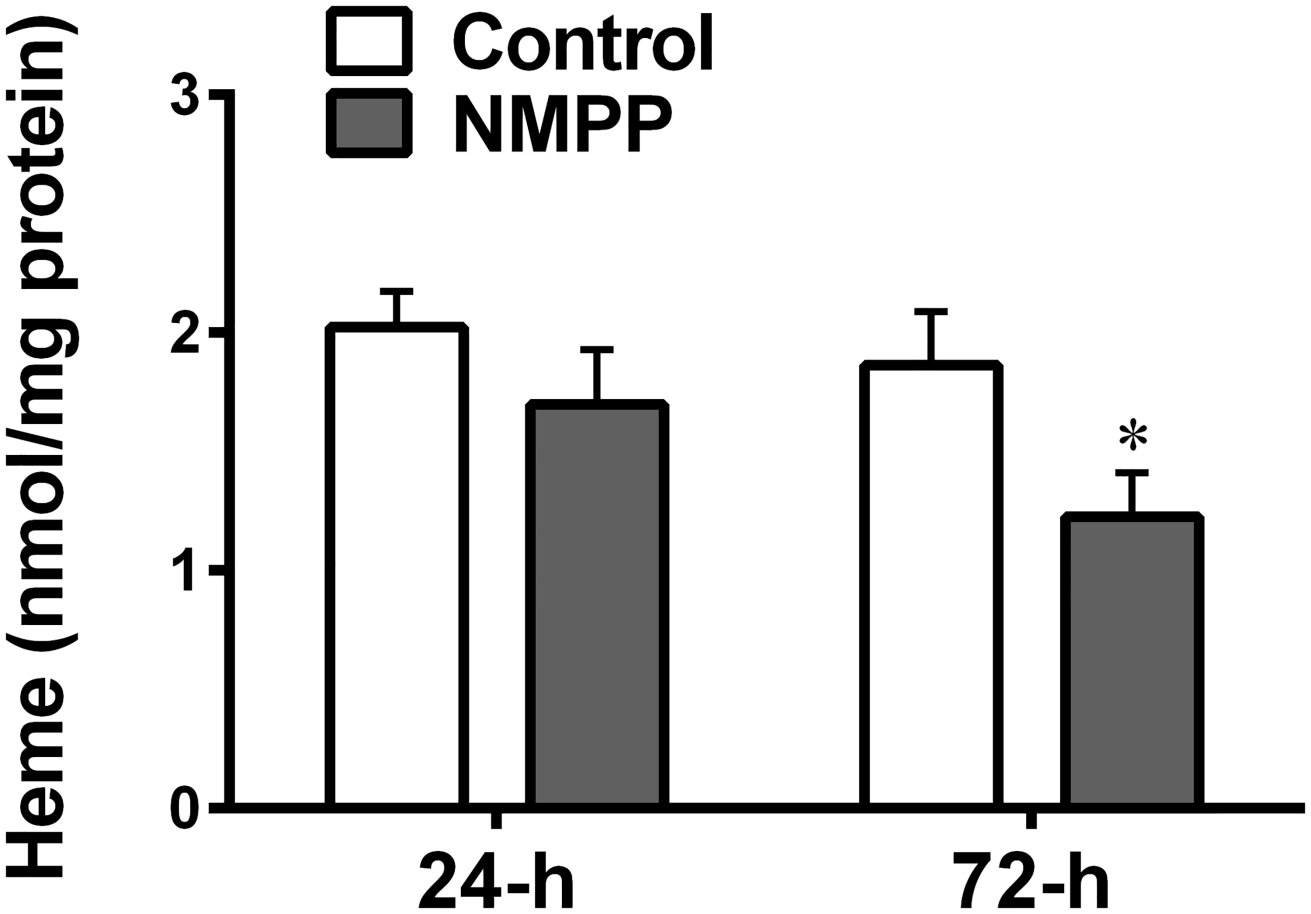
Changes in vascular heme levels in BCAs cultured with NMPP (10^-6^M) for 24 and 72 hours respectively (n = 12). *significant difference from controls.

Fig 1a shows that the incubation of BCAs with NMPP for 24 hours (24-h) significantly reduced their FECH activity in a dose-dependent manner, compared to control vessels. A time-dependent inhibition of FECH activity by NMPP was also revealed, as indicated by the result that at each concentration point, the reduced FECH activity was significantly greater in BACs treated with NMPP for 72-hour (72-h) than those of 24-h. Additionally, 10^-7^M NMPP, did non-statistically reduce FECH activity in the first 24 hours (P = 0.096), however, a statistically significant inhibition was observed after extending incubation period to 72 hour (p = 0.031). Because our preliminary results showed that the high dose of NMPP (10^-5^M) elicited an impaired vasoconstrictor response after treating BCAs for 24-hour, whereas an effective inhibitory effect initiated by the low dose of NMPP (10^-7^M) required a longer treating period. To this end, 10^-6^M NMPP was selected as an optimal dose used in following experiments in order to ensure its pharmacologically sufficient activity without interrupting with vascular contractility. As shown in Fig 1b, in the presence of 10^-6^M NMPP, protein expression of FECH was not significantly affected in the either group of vessels, suggesting a post-translational inhibition of FECH. Fig 2 shows a time-dependent reduction in BCA heme levels in response to NMPP treatment, suggesting the heme synthesis in a FECH-dependent manner.

### NMPP reduces endothelium-dependent and –independent relaxation of BCAs via impairing NO/sGC/VASP pathway

**Fig 3:**
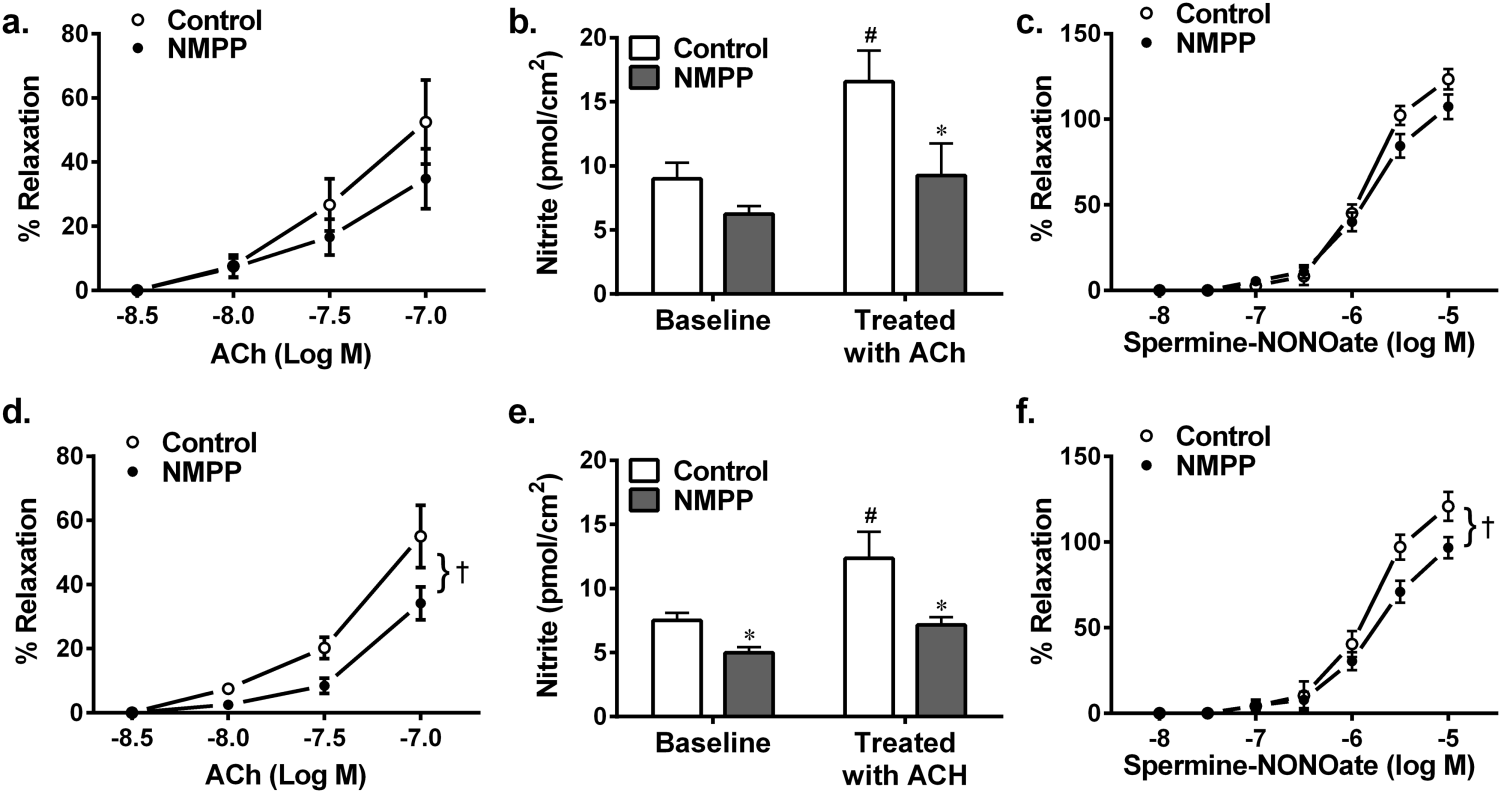
Changes in vascular relaxations to Acetylcholine (ACh; a & d) and spermine-NONOate (c & f), as well as basal and stimulated release of nitrite/NO (b & e) in BCA rings cultured with NMPP (10^-6^M) for 24 (a-c) and 72 (d-f) hours respectively. (n = 12–15). *significant difference from their controls. #significant difference from baseline controls. † significant difference between the two curves.

**Fig 4:**
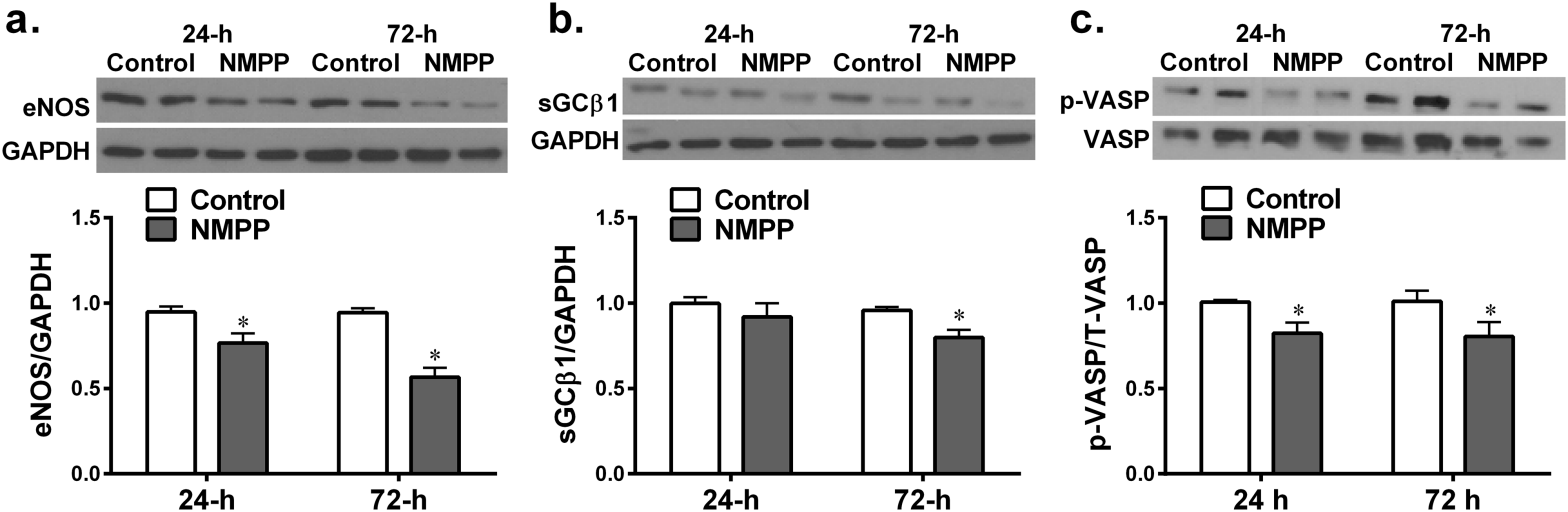
Changes in protein expressions of endothelial nitric oxide synthase (eNOS; a), soluble guanylyl cyclase-subunit 1 (sGCβ1; b) and phosphorylation of vasodilator-stimulated phosphor-protein (p-VASP; c) in BCAs cultured with NMPP (10^-6^M) for 24 and 72 hours respectively. (n = 5 blots for each group). *significant difference from their controls.

**Fig 5:**
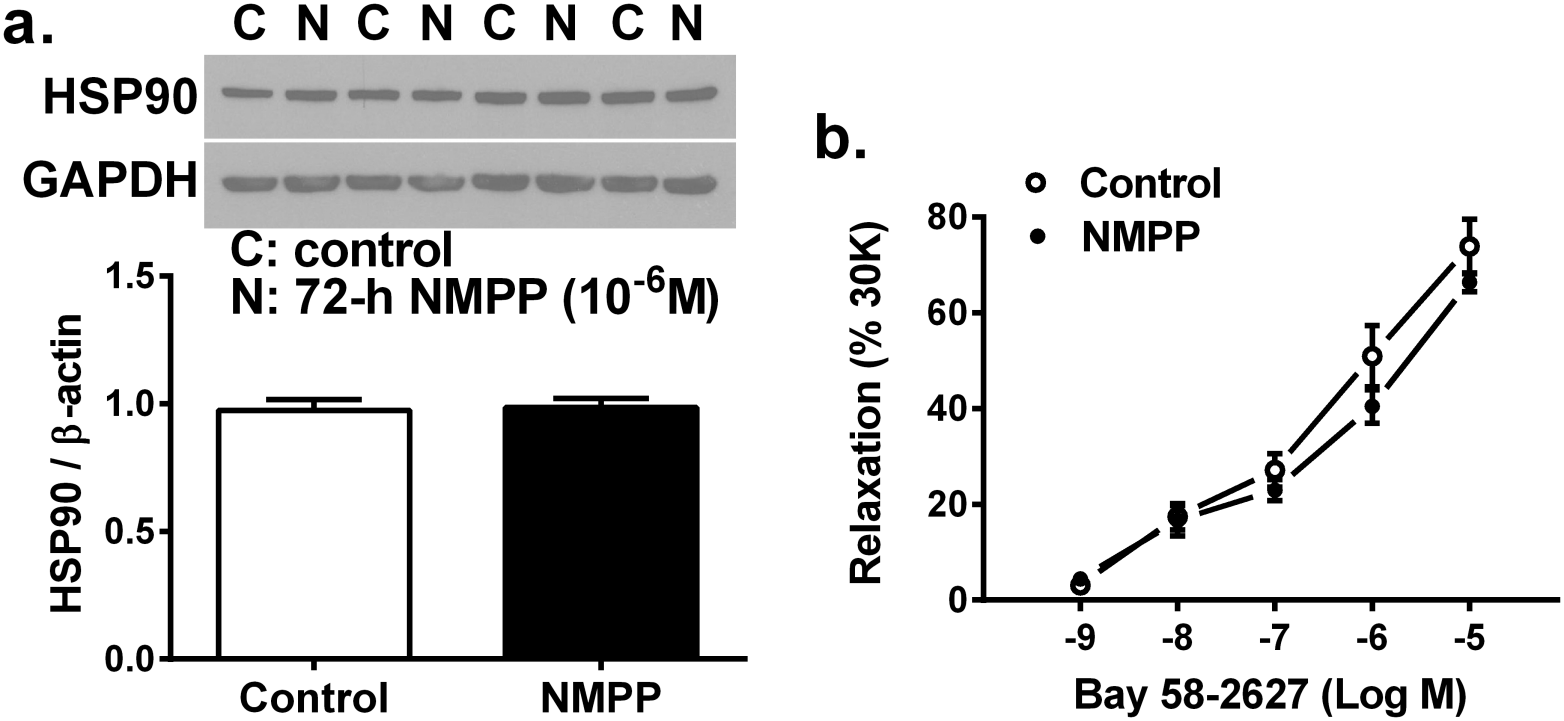
Vascular protein expression of HSP90 (a; n = 8 for each group) and relaxation response to BAY58-2627 (b; n = 8 for each group) in BCAs in the control condition and cultured with NMPP (10^-6^M) for 72 hours.

Next, functional evidence of the reduction in FECH activity is depicted in Fig 3. As indicated, treatment of BCAs with NMPP for 72 hours (d-f) significantly attenuated ACh-induced relaxations (d), concomitantly associated with significant reductions in both basal and ACh-stimulated release of nitrite/NO (e), verifying the failure of sufficient release of endothelial NO leading to the impaired endothelium-dependent relaxation. Moreover, NMPP-treated BCAs for 72-h displayed also, significant declines of spermine-NONOate (NO donor)-induced relaxations (f), suggesting impaired endothelium-independent responses attributed to reduction in NO activating sGC. A similar responsive pattern was also presented in the group treated with NMPP for 24 hours (a-c), which however, did not reach statistical significance (p = 0.15 and p = 0.07 by two-way ANOVA for ACh and NONOare-induced dilations, respectively), implying that a time-dependent treatment was required to observe this action of NMPP. Thus, the inhibition of vascular FECH (Fig 1) sequentially followed by the decrease in heme production (Fig 2) appears to alter the heme-containing synthase enzymatic activities of eNOS and sGC, leading to impaired NO-release and -mediated responses (Fig 3). This conclusion was verified by data in Fig 4 showing that the inhibition of FECH with NMPP directly suppresses the protein expression of eNOS (a) and sGC (b). Using phosphor-VASP (at Ser^239^) as an index of upstream-located sCG activation confirms a downregulation of p-VASP expression being as a consequence of sCG inactivation (Fig 3c). Alternatively, NMPP that inhibited heme synthesis to downregulate sGC did not significantly affect HSP90 expression (Fig 5a), neglecting the possibility of suppression of sGC expression attributed to an altered expression of HSP90, which in turn, leads to failure of inserting heme group into the enzyme [24,25]. Moreover, in order to verify the impaired sGC-mediated relaxation (Fig 3) and sGC downregulation (Fig 4) being heme-dependent in nature, separate experiments were performed aimed to assess vascular relaxation to Bay 58–2627, a vasoactive agent that specifically activates heme-depleted form of sGC [26,27]. As shown in Fig 5b, Bay 58–2627 elicited a comparable dose-dependent relaxation in the control and NMPP-treated BCAs, revealing a normal vascular response to the heme deficient component of sGC.

### Effects of NMPP on BCA responses to non-NO responsive agents

**Fig 6:**
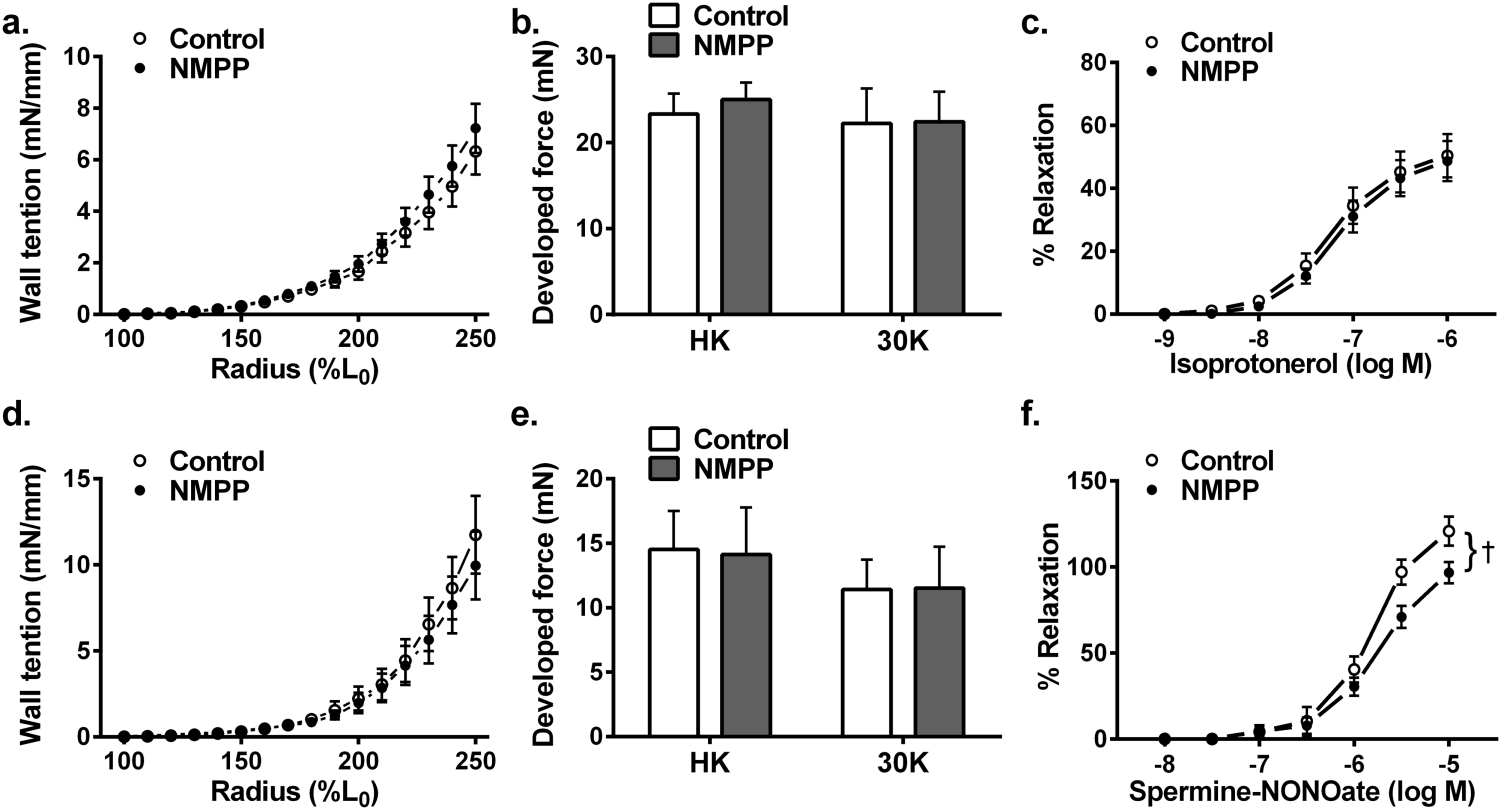
Vascular wall tension in response to stretch-induced changes in radius (a & d), force development in response to stimulation with 122 mM KCl (HK) and 30 mM KCl (30K) (b & e), and vascular relaxations to isoproterenol (c & f) in BCA rings cultured with NMPP (10^-6^M) for 24 (a-c) and 72 (d-f) hours respectively. (n = 11–14).

To document the specificity originating from changes in eNOS/NO/sGC/cGMP signaling as a function of inhibiting FECH/heme by NMPP, the mechanical properties of BCAs were determined by assessing their length-tension relationship and vascular responses to NO/sGC-independent mediators. Fig 5 summarizes that the control and NMPP-treated BCAs exhibited an identical response to the mechanical stretch, as indicated by their overlapped length-tension curves (a & d). Additionally, vascular contractions manifested by the force development to 122 mM and 30 mM KCL respectively, (b & e) and relaxations to isoproterenol (c & f; β-adrenergic receptor activator of cAMP) were comparable between NMPP-treated and –untreated groups of BCAs. These results indicate that NMPP-induced changes (Figs 1–3) impact specifically on vascular FECH/heme/eNOS/sGC signaling. Moreover, NMPP did not elicit changes in BCA superoxide production (Fig 6), suggesting that the observed changes in vascular function caused by the NMPP-inhibition of FECH are independent of superoxide production.

## Discussion

We provided direct evidence indicating that eNOS and sGC are two independent targets for NMPP that inhibits FECH-dependent heme synthesis to downregulate eNOS and sGC expression, leading to impaired NO/sGC/cGMP-mediated vasodilator responses, which are independent of vascular oxidative stress. Although accumulating evidence has indicated an oxidation-dependent alteration of FECH/heme/sGC signaling in vasculatures, characterized as impaired NO-mediated responses [12,16,18,24,28–30], two important points have not been unaddressed yet, as what is direct effect of deleting FECH/heme on eNOS expression, and 2) whether an alternative mechanism independent of oxidative stress is involved. Thus, the present study was focused on direct correlation between the FECH/heme and two heme-dependent enzymes, via assessing eNOS and sGC protein expression and activity, as a function of NMPP-inhibition of FECH/heme without interruptions from oxidative stress.

### Inhibition of vascular FECH/heme/eNOS/sGC/cGMP/relaxation by NMPP

We found that chronic incubation of BCAs with NMPP elicited a dose- and time-dependent reduction in FECH activity, without significant changes in FECH protein expression (Fig 1), suggesting a posttranslational-based event of NMPP, via most likely, instable of iron-sulfur cluster assembly machinery of FECH [6]. As a result of inhibitory FECH activity, downstream production of heme was concomitantly attenuated in a time-dependent manner (Fig 2), revealing a FECH-specific suppression of heme biosynthesis. The dose of 10^-6^M NMPP was selected because of its sufficiently inhibiting NMPP activity and heme production without affecting normal vascular responsiveness. Functional changes in response to NMPP-induced reductions in FECH activity and heme synthesis were evidenced by Fig 3 showing that after treatment of BCAs for 72 hours, endothelium-dependent ACh-induced vasodilation and ACh-stimulated vascular release of NO were significantly attenuated. Moreover, endothelium-independent relaxation by an exogenous NO donor that directly targets and activates sGC on vascular smooth muscle to elicit cGMP-mediated relaxation, was also significantly reduced. Thus, as downstream targets of FECH/heme, both eNOS and sGC are separately affected by NMPP via inhibiting FECH to block heme synthesis. Noteworthy, functional alterations of vessels (Fig 3) are correspondingly matched with changes in the heme level (Fig 2) and FECH activity (Fig 1) as a function of NMPP, expressed as progressively declining relaxation during 24- to 72-hour incubation period, confirming the relationship among FECH, heme, eNOS, sGC and cGMP in the signal transduction. In consistence with currently published findings that FECH siRNA prevents ocular neovascularization via deleting FECH-induced angiogenesis and also partially, suppressing eNOS expression [31], we indicated a direct downregulation of eNOS expression (Fig 4a), accompanied with suppressing sGC protein expression (Fig 4b) by NMPP, suggesting that while NMPP interferes with FECH activity at a post-translational level (Fig 1), it initiates a post-transcritipnal downregulation of eNOS and sGC due perhaps, to the result of deficient synthesis of heme (Fig 2). In this context, we provided explanations for reductions in systemic nitrite/nitrate excretion and renal NOS and sGC activities, associated with altered endothelium-dependent and –independent relaxations of aorta in response to *in vivo* deleting heme synthesis of rats [19]. Of note, the significant downregulation of eNOS in BCAs treated with NMPP for 24 hours (Fig 4a) elicited corresponding reductions in the ACh-stimulated release of NO (Fig 4b), while vascular relaxation to ACh appeared to be preserved (Fig 3a). This phenomenon may attribute to the ACh compensatory release of non-NO mediator(s), as a function of insufficient NO production, to maintain vascular relaxation [32]. Alternatively, the downregulation of sGC expression in response to NMPP treatment (Fig 4b) did reflect corresponding impairment in its activity (Fig 3c). Consistently, a significant suppression of vascular VASP phosphorylation by NMPP-treatement (Fig 4c) confirms the FECH inhibition-dependent inactivation of sCG. Moreover, sufficient synthesis/level of heme is essential for the maintenance of normal sGC function, which has been demonstrated in our studies indicating a NMPP-induced reduction of heme synthesis, leading to the damage of sCG function (Figs 1–4). Additionally, heme insertion also plays key roles during maturation of sGC, a response that is driven by HSP90 [25,33], and inhibition of HSP90 is able to attenuate sGC activity via increasing its degradation [34]. In our studies, the impaired sGC activity was independent of HSP90-mediated pathway because NMPP did not change HSP90 expression (Fig 5a) and activation of HSP90–associated heme-deficient (apo) sGC by BAY58-2627 remained normal in NMPP-treated BCAs (Fig 5b). This confirms our conclusions that the impaired sGC is driven directly by NMPP inhibition of heme production.

### NMPP-elicited changes in FECH/heme/eNOS/sGC/cGMP signaling independent of oxidative stress, changes in vascular property and cAMP-mediated pathway

**Fig 7:**
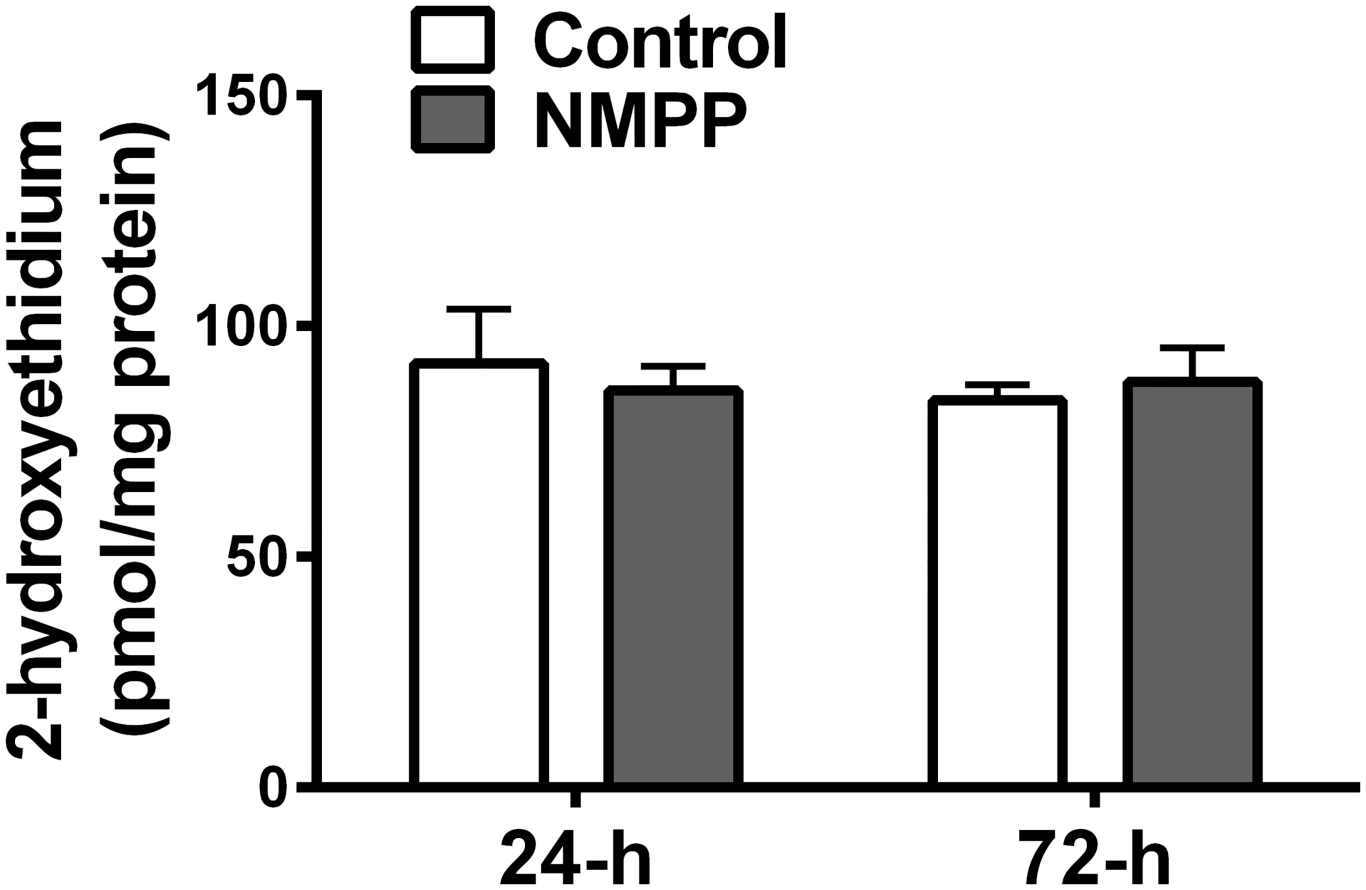
Vascular superoxide production, indicated by 2-hydroxyethidium levels in BCAs cultured with NMPP (10^-6^M) for 24 and 72 hours respectively. n = 8.

As demonstrated (Fig 6), neither vessel responses to mechanical stretch (a & d) or smooth muscle depolarization by high potassium (b & e), nor isoproterenol-induced cAMP-mediated relaxation (c & f) was affected by NMPP treatment, verifying the specificity of NMPP-inhibition of vascular FECH/heme/eNOS/sGC/cGMP signaling. On the other hand, as a key player in the mediation of cardiovascular dysfunction, oxidative stress potentially insults heme biosynthesis, via perhaps, disrupting FECH Fe-S cluster [7], a specific structure that is indispensable for maintaence of FECH stability and activity [6]. The consequences arising therefrom, are characterized as the attenuation of heme-containing enzyme synthesis. Indeed, the one of mechanisms responsible for oxidative stress-dependent deletion of sGC is considered to be due to an oxidation of sGC heme moiety-induced heme detachment from the enzyme, the response that facilitates degradation of the enzyme by the ubiquitin-proteasome proteinlysis pathway [35,36]. Moreover, the oxidant-dependent inactivate NO bioavailability has been well documented [37]. Alternatively, aimed to identify a direct inhibition of FECH by NMPP in an oxidative stress-independent manner, the present study was conducted on a physiologically-based *in vitro* condition. The well preserved vascular responsiveness (Fig 6) and unchanged vascular superoxide level (Fig 7) in BCAs treated with NMPP up to three days verify a specific feature of the study, which differs from our, as well as others’ previous studies showing primarily a superoxide-dependent deletion of sGC activity [9,10,16,18,29].

In conclusion, we provided direct evidence for the deletion of vascular heme production, as a function of inhibiting EFCH without interference from oxidative stress, elicit deficiency of downstream-located eNOS and sGC synthesis and activity, leading to impaired coronary vasodilator responses specifically mediated by cGMP. Our findings shed light on the physiological significance of FECH/heme involved in essentially operating vascular function. In addition, during some pathological processes, FECH may serve as a therapeutic target with its activator(s) to favor and/or preserve eNOS/sGC/cGMP-mediated vascular functions.

## Supporting information

S1 Experimental Data (XLSX)
